# Heavy and Chronic Cannabis Use Impact on Human Emotions: BOLD-fMRI Study

**DOI:** 10.1192/j.eurpsy.2023.1936

**Published:** 2023-07-19

**Authors:** S. Boujraf, M. Jaafari, A. Houate, R. Aalouane, M. Maaroufi, I. Rammouz

**Affiliations:** Clinical Neurosciences Laboratory, Faculty of Medicine, Fez, Morocco

## Abstract

**Introduction:**

Long term cannabis use has been expanding drastically over the last two decades and has become a major health issue worldwide. Recent studies demonstrate that brain complications in adults with cannabis use are associated with cognitive and emotional impairments, but little is known about the relationship between structural alterations and behavioral manifestations. Therefore, studying the relationship between alterations of emotional system, in parallel with structural degenerative phenomena is very critical.

**Objectives:**

Hence, the aim of this study is to demonstrate such alterations by making use of appropriate paradigms during BOLD-fMRI scans. Positive, negative and neutral emotions were examined, in relations with DTI and functional connectivity.

**Methods:**

11 cannabis addicted patients volunteered for the study. Volunteers were fully healthy. However, any additional comorbidity was a strict criterion of exclusion, and a healthy general state was an indispensable criterion of inclusion. Additionally, it was excluded any patient that have any additional substance use such as tobacco, alcohol, cocaine, etc. And strict use of cannabis was a must.

All patients underwent blood and urine assessments to ensure the selection criteria.

All patients underwent BOLD-fMRI and anatomical MRI using both motor and emotional paradigm.

The motor task consisted of rest alternating with finger tapping. The emotional task included 3 conditions. Positive, neutral and negative were each alternating with silent mental counting. The fMRI data was processed using SPM12 package. A sample of 12 age-matched controls was also included.

**Results:**

The present results are based on analysis of behavioral and BOLD-fMRI data of 11patients and similar age-matched controls. Analysis of behavioral data showed an alteration of emotional abilities in cannabis addicted patients compared to controls. Analysis of fMRI data revealed significant changes of activation within a large cortical network including prefrontal cortex and parietal cortex, and that emotional responses and BOLD signal were inversely correlated.

**Conclusions:**

These findings demonstrate that the brain of cannabis addicted patients undergoes and emotional alterations that parallel silent structural degenerative phenomena. Although the causal mechanisms are still to be investigated, the fact that functional impairments can be detected in emotional, cognitive and motor domains calls for the development of preventive measures using neurobehavioral tools for this population of patient, and even in at risk users.

**Disclosure of Interest:**

None Declared

